# Alexithymia and cortisol awakening response in people with eating disorders

**DOI:** 10.1192/j.eurpsy.2021.326

**Published:** 2021-08-13

**Authors:** G. Cascino, A.M. Monteleone, F. Marciello, V. Ruzzi, F. Pellegrino, P. Monteleone

**Affiliations:** 1 Department Of Medicine, Surgery And Dentistry, University of Salerno, Baronissi/Salerno, Italy; 2 Department Of Psychiatry, University of Campania “Luigi Vanvitelli”, Naples, Italy

**Keywords:** eating disorders, alexithymia, cortisol, stress

## Abstract

**Introduction:**

Alexithymia, that is the inability to recognize and describe one’s own emotions, is a transdiagnostic feature across eating disorders (EDs) and it has been associated to a prolonged stress exposure.

**Objectives:**

Therefore, we evaluated whether alexithymia affects the hypothalamus-pituitary-adrenal (HPA) axis functioning in patients with anorexia nervosa (AN) or bulimia nervosa (BN).

**Methods:**

Twenty-six women with AN and 26 with BN participated in the study. Alexithymia was evaluated by the Toronto Alexithymia Scale–20 and eating-related psychopathology was measured by the Eating Disorder Inventory-2. The activity of the HPA axis was assessed by the salivary cortisol awakening response (CAR). Group differences in saliva CAR were tested by repeated measures 3-way ANOVA with diagnosis and alexithymia as between-subject factors.

**Results:**

The prevalence of alexithymia did not differ significantly between the two diagnostic groups (c^2^=1.24, p=0.26). Alexithymia was associated with more severe eating-related psychopathology in AN women but not in BN women. A significant reduction in the magnitude of CAR occurred in alexithymic patients with BN compared to non-alexithymic patients with BN (t = 3.39, p = 0.008), but not in alexithymic women with AN (t = 0.67, p = 0.54).

**Conclusions:**

These results confirm the presence of a more severe eating-related psychopathology in alexithymic individuals with AN and show, for the first time, an association between alexithymia and a dampened basal activity of the HPA axis in BN.

**Disclosure:**

No significant relationships.

